# The differential roles of shame and guilt in the relationship between self-discrepancy and psychological maladjustment

**DOI:** 10.3389/fpsyg.2023.1215177

**Published:** 2023-09-28

**Authors:** Hyunjoo Oh, Dong-gwi Lee, Hunggu Cho

**Affiliations:** ^1^Department of Psychology, Yonsei University, Seoul, Republic of Korea; ^2^Counseling and Psychological Services, College of Education and Human Development, Atlanta, GA, United States

**Keywords:** self-discrepancy, shame, guilt, depression, anxiety

## Abstract

The self-discrepancy theory proposes that having inconsistent self-representations can trigger feelings of shame and guilt, leading to experiences of depression and anxiety. The aim of this study was to determine the distinct characteristics of each shame and guilt in relation to the connection between actual/ideal self-discrepancy and depression, as well as actual/ought self-discrepancy and anxiety. A total of 403 participants completed an online questionnaire assessing their self-discrepancy, shame, guilt, depression, and anxiety. Correlational analysis and structural equation modeling (SEM) analysis were used to assess the goodness of fit of the proposed model and the structural relationships between the variables. The key findings were as follows: (1) There were positive correlations among actual/ideal self-discrepancy, actual/ought self-discrepancy, shame, guilt, depression, and anxiety; (2) Shame partially mediated the association between actual/ideal self-discrepancy and depression; and (3) Guilt fully mediated the association between actual/ought self-discrepancy and anxiety. These outcomes uphold the self-discrepancy theory by confirming a distinct intra-psychological process involving shame and guilt. Each type of self-discrepancy was related to experiences of depression and anxiety. Our data suggest that researchers and practitioners should prioritize shame and guilt when examining individuals’ self-discrepancy and related mental health challenges.

## Introduction

The transition from adolescence to young adulthood often brings about a range of developmental challenges, encompassing both social and psychological aspects. These challenges include establishing personal identity and autonomy, forming interpersonal relationships, and navigating career development (Biasi et al., 2017). To describe this distinct period of development spanning from ages 18 to 29, Arnett (2000) introduced the term “emerging adulthood.” This phase, as argued by Arnett (2000, 2011) is particularly reflective of the millennial generation (late teens to twenties) in Korea, setting it apart from what might be termed “extended adolescence.” During this time, young adults, having gained independence from parental control, embark on individual explorations in terms of both career choices and social adaptation (Arnett, 2000).

Arnett (2004a,b) proposed that emerging adulthood is marked by five distinct characteristics; (a) identity exploration, (b) feeling in-between, (c) experimentation and possibilities, (d) self-focus, and (e) instability. This characterization emphasizes the heightened susceptibility to significant psychological challenges during this developmental phase (Kim and Kim, 2020). Notably, depression and anxiety stand as prominent mental health issues among Korean undergraduate students (An and Hong, 2022). These conditions are also closely linked to other severe psychological maladjustments, including interpersonal problems and alcohol abuse (Weitzman, 2004; Triscoli et al., 2019). As such, depression and anxiety are considered overarching factors that encompass a wide spectrum of mental health issues among emerging adults (Bamber and Morpeth, 2018).

A national mental health survey reveals that, due to the prolonged impact of the COVID-19 pandemic, young adults have reported the highest levels of severe anxiety and depression, resulting in a significant decline in their mental well-being in comparison to middle-aged adults in South Korea (Lee and Kim, 2022). Gender differences also emerge, with women reporting higher levels of depression and anxiety symptoms than men. Notably, the most prominent prevalence of mental health risk groups has been identified among young adults in their 20s and 30s. For those in their 20s, the rates of depression and anxiety stand at 20.22 and 8.89% respectively, while those in their 30s show rates of 26.99% for depression and 13.11% for anxiety. Similarly, a comprehensive review of epidemiological studies conducted in the United States underscores that individuals aged 18 and 29 exhibit a prevalence of over 40% for any psychiatric disorder, surpassing rates seen in other age groups (Korean Ministry of Health and Welfare, 2022). This discrepancy is particularly pronounced for conditions like anxiety disorders, mood disorders, and substance abuse problems. Furthermore, recent studies have reported that the spread of the COVID-19 virus affects the psychological well-being of individuals with emotional experiences of shame and guilt (Haller et al., 2020; Mayer and Vanderheiden, 2021), which exacerbate the severity of traumatic symptoms of COVID-19-related events (Cavalera et al., 2023). In terms of the link between these self-conscious emotions and psychological distress during the pandemic, people are likely to engage in negative self-evaluation following infection, with a greater likelihood of feeling stigmatized, denied, ashamed, and depressed. In addition to shame, individuals experience intense feelings of fear, worry, and guilt for potentially infecting others (Li et al., 2020; Hamama and Levin-Dagan, 2022).

Researchers have mainly focused on intrapersonal characteristics such as cognitive-content variables (e.g., irrational beliefs, self-discrepancy, and maladaptive perfectionism) that contribute to depressive and anxiety symptoms (Rice and Ashby, 2007; Chan and Sun, 2021). According to the cognitive model perspective, the self-concept encompasses cognitive constructs about oneself (Sedikides and Skowonski, 1997) that can impact cognitive, affective, and behavioral outcomes (Bong and Skaalvik, 2003). In this context, the perceived self-discrepancy used in this study pertains to the cognitive aspect (Festinger, 1957). Such incongruity in self-state representation amplifies the activation of negative self-perception, giving rise to various psychological maladjustments (Lewis, 1971; Higgins et al., 1986).

Previous literature has examined the relationship between self-discrepancy and negative emotions or psychological difficulties, mainly focusing on the links between the variables of interest (Strauman, 1989; Jung and Hong, 2016). For example, many studies have examined the association between actual/ideal self-discrepancy, shame, and depression (Jo and Jung, 2015; Sonnenburg and Miller, 2021). However, the other dimension of self-discrepancy, namely actual/ought self-discrepancy, and the corresponding experiences of guilt and anxiety have not been simultaneously considered. Similarly, some studies have examined the relationship between actual/ought self-discrepancy, anxiety, and guilt (Park and Chang, 2017; Dong, 2020; Zhang et al., 2021), neglecting the inclusion of actual/ideal self-discrepancy and its associated psychological symptoms (i.e., shame, depression). This approach limits a comprehensive understanding of how shame and guilt differentially mediate the process by which each type of self-discrepancy relates to depression or anxiety within a unified research model.

Importantly, there is evidence to suggest that inconsistent findings within the same theoretical framework may result from differences in how researchers define concepts and selectively include variables of interest in the research model, despite the need to consider other relevant variables to test or evaluate a particular theory (Agnew, 2015; Park and Bae, 2016). Such an approach has limited the ability to predict the affect-specific relationships between different self-discrepancies and depression or anxiety, as well as the role of shame and guilt in these processes. For instance, Liss et al.’s (2013) study examined self-discrepancy, shame, guilt, and fear of negative evaluation, but was limited to ‘mothers’ and measured only actual/ideal self-discrepancy. Similarly, Castonguay et al. (2012) explored self-discrepancy and self-conscious emotions, but their focus was limited to actual/ideal self-discrepancy.

Given the relative paucity of research investigating actual/ought self-discrepancy and guilt or anxiety, compared to that investigating actual/ideal self-discrepancy and the related variables of shame and depression (Stevens et al., 2015), the theoretical and empirical validation of the self-discrepancy theory has been limited without fully unfolding the dynamics of self-discrepancy in relation to psychological distress. Therefore, a more comprehensive and integrative examination of research models based on a specific theoretical framework is essential, and our current study provides an alternative to overcome limitations. Within a single research model based on this theoretical framework, we examine the distinct roles of shame and guilt as mediators in the processes of (1) actual/ideal self-discrepancy and depression, and (2) actual/ought self-discrepancy and anxiety.

In the self-discrepancy theory, Higgins (1987) posits that different self-discrepancies are related to different types of psychological vulnerability. Specifically, discrepancies between actual and ideal self-images are associated with *dejection-related emotion* (e.g., sadness, frustration, and disappointment), which corresponds to the absence of positive outcomes, such as love and approval. In contrast, conflicts between actual and ought self-state representations are associated with emotional discomfort in the presence of negative results, triggering *agitation-related emotion* (e.g., tension, guilt, and fear). In accordance with Higgins’s proposition, a sizable body of research has supported distinct associations between different self-discrepancies and psychological problems (Higgins et al., 1986; Strauman, 1989; Scott and O'Hara, 1993); however, other researchers have returned contrasting results. For example, a recent meta-analysis revealed that actual/ideal and actual/ought self-discrepancies were related to both depression and anxiety (Mason et al., 2019). Other researchers have found that actual/ideal self-discrepancy is associated with both depressive and anxiety symptoms, whereas actual/ought self-discrepancy is only associated with anxiety symptoms (Dickson et al., 2019). Given the large gap in understanding the relationship between specific self-discrepancies and depression and anxiety, it is necessary to explore the underlying intra-psychological processes in which mismatches in self-concept may lead to specific psychological distress (Boldero et al., 2005).

Shame and guilt are self-conscious and secondary emotions associated with more complicated cognitive processes, such as self-reflection and self-assessment, than primary emotions, including basic and immediate responses (e.g., fear of threatening situations and sadness after a loss; Lewis et al., 1992; Tracy and Robins, 2007). These emotions rely on a sophisticated cognitive apparatus demanding advanced self-evaluative skills (Lewis et al., 1992).

While the terms shame and guilt have often been used interchangeably (Dearing et al., 2005), extensive theoretical work has established precise conceptual and functional distinctions between these emotions (Tangney et al., 2007; Zhu et al., 2019). Lewis (1971) highlighted “the self” in differentiating between shame and guilt. Specifically, different foci of devaluation are critical in delineating shame and guilt and the existence of the self vs. the behavior of the self. People who experience shame are likely to direct their focus inward, and “the entire self” becomes a target for devaluation (the “bad self”). They perceive themselves as “small,” “defective,” and “worthless,” especially when they fail to achieve their ideal self-presentation. Experiencing shame also comes with the feeling of being exposed, leading to behavioral withdrawal (Sabini and Silver, 1997). For the experience of guilt vis-à-vis shame, the localization of negative valuation originates in specific behaviors or a lack thereof. Thus, in a guilt state, certain activities of the self become an object of reproach (Schmader and Lickel, 2006), resulting in more “approaching” phenomena (e.g., apology, reparation, confession) to rectify devalued behaviors (Frijda et al., 1989). Although people may feel bad for themselves when they engage in offensive behavior, their sense of global identity is retained, unimpaired, and integrated (Lindsay-Hartz, 1984; Niedenthal et al., 1994). Because the target of negative evaluation is specific behavior, one’s sense of global identity is retained, unimpaired, and integrated (Lindsay-Hartz, 1984; Niedenthal et al., 1994). Thus, shame is more likely to be painful than guilt, as it strikes at the heart of one’s identity (Tangney, 1993).

According to Higgins’s self-discrepancy theory, shame is associated with a mismatch between perceived actual/ideal selves, whereas guilt can result from actual/ought self-discrepancy. Prior studies have revealed that shame and guilt are related differently to specific psychological problems (Orth et al., 2006; Jeong and Shin, 2014); however, the association between guilt and anxiety remains poorly understood (Song, 2008; Gürcan-Yıldırım and Gençöz, 2020). Supporting the self-discrepancy theory (1987), the process model of self-conscious emotions illustrates that shame and guilt emerge when a widening gap between one’s actual and ideal or ought self-states is perceived (Tracy and Robins, 2004). Being aware of this gap can lead to tension and feelings of being flawed, inadequate, and sinful (Piers and Singer, 1971; Wurmser, 1981; Morrison, 1989; Barnett et al., 2017).

However, it is important to note that negative emotions like shame and guilt are not always accompanied by self-discrepancy (Petrocelli and Smith, 2005; Chung and Cho, 2018). Not everyone experiencing self-discrepancy will necessarily feel shame or guilt. This raises questions about potential moderating variables that may explain when and for whom self-discrepancy triggers these negative emotions (Higgins, 1999; Boldero et al., 2005). For example, individuals with a high fear of social evaluation exhibit a strong positive link between self-discrepancy and shame, whereas those with low fear of negative evaluation from others do not exhibit the same pattern. This suggests that the fear of being judged in social situations could moderate the relationship between self-discrepancy and negative emotion of shame (Liss et al., 2013). Additionally, emotional clarity acts as a possible protective factor, moderating the relationship between actual/ideal self-discrepancy and shame (Won and Kim, 2019).

Consequently, this study investigates the relationships between different types of self-discrepancy and two indices of psychological problems (depression and anxiety). We also examine how shame and guilt function differently during this process. Thus, we hypothesize that (1) shame mediates the relationship between actual/ideal self-discrepancy and depression (Hypothesis 1) and (2) guilt mediates the relationship between actual/ought self-discrepancy and anxiety (Hypothesis 2).

## Methods

### Participants and procedure

A total of 403 Korean college students (*190* men, *213* women) aged 19 or over participated in this study (*M* = 21.93, *SD* = 1.82). Data were collected via an online survey platform, dataSpring. The participants provided informed consent online before starting the survey. Those who completed the survey were rewarded with points that were converted into cash. All research methods and procedures were approved by the university’s institutional review board (IRB approval number: 7001988-202102-HR-990-06).

### Measures

Self-discrepancy was measured using Seo’s (1996) adaption of the Selves-Questionnaire, originally developed by Higgins et al. (1985). It consists of 22 opposite adjectival pairs that consider three domains of the self (actual, ideal, and ought) from two perspectives (own and other). Here, we consider the three domains in relation to the self and self-view. The questions were rated using 9-point Likert-type scales with a center point of 0 (0 = not at all; 4 = strongly agree). The internal consistency reliability estimates (Cronbach’s alpha) for the actual, ideal, and ought selves were 0.87, 0.90, and 0.93, respectively (Seo, 1996), and those for this study were 0.88, 0.95, and 0.95, respectively. Among the 22 total items, 15 items in the “evaluative factor,” 2 items in the “potency factor,” and 2 items in the “active factor” exhibited distinctly high factor loadings and the other 3 items showed similar levels of factor loadings in both the evaluative and potency factors, establishing convergent validity (Osgood et al., 1957).

The Test of Self-Conscious Affect (TOSCA-3; Song, 2008), originally developed by Tangney et al. (1989), was used to measure shame and guilt. Using a scenario-based approach, the TOSCA-3 consists of 16 questions to which participants respond with what they would do in a series of everyday situations, followed by responses including shame, guilt, detachment/unconcern, externalization, and alpha and beta pride. Responses to each scenario were rated using a 5-point Likert scale (1 = not likely; 5 = very likely). In this study, items related to shame and guilt were used. The internal consistency of the TOSCA-3 was 0.81, and shame and guilt were 0.78 and 0.70, respectively (Song, 2008). In this study, the internal consistency was 0.85 for shame and 0.79 for guilt. The scenarios related to each shame and guilt showed convergent validity and construct validity. Also, both emotions were related but, distinct from each other, supporting the discriminant validity (Lacerenza et al., 2020).

The Center for Epidemiological Studies Depression Scale (CES-D; Radloff, 1977) is widely used to measure depression. The Korean version of the CES-D, compiled by Chon et al. (2001), was used to measure depression. It consists of 20 items rated on a 4-point Likert scale (0 = almost never; 3 = almost all of the time). The internal consistency of the Korean version of the scale was 0.91 for Chon et al. (2001) and 0.92 for this study. Also, the results of factor analysis showed that a four-factor solution had an acceptable fit with 58.7% of the variability (Chon et al., 2001).

Anxiety was assessed using the Korean version of the State–Trait Anxiety Inventory (K-STAI; Hahn et al., 1996), developed from the original version of the STAI-Y (Spielberger, 1983). The K-STAI includes 40 items, 20 of which assess traits and the state of anxiety. Only 20 items assessing the state of anxiety were included in this study. Participants indicated whether they felt anxious using a 4-point Likert scale (1 = strongly disagree; 4 = strongly agree). The internal consistency reliability estimate of the K-STAI was 0.92 in Hahn et al.’s (1996) study, and 0.94 in this study. Furthermore, the concurrent validity of the K-STAI with the Manifest Anxiety Scale (MAS; Taylor, 1953), which measures anxiety as a personality trait and the Maudsley Medical Questionnaire (MMQ, Eysenck, 1952) was found to be 0.50 and 0.45, respectively (Hahn et al., 1996).

### Data analysis

Statistical analyses were conducted to examine the structural relationships among self-discrepancies (actual/ideal and actual/ought), shame, guilt, depression, and anxiety using structural equation modeling (SEM) in the AMOS program. As the “unidimensionality” of all variable structures is established, the item parceling approach was adopted to test the proposed model (Russell et al., 1998; Bandalos, 2002). Following the suggestion that subgroups are formed through item parceling by calculating the mean scores of each item (Little et al., 2013), we parceled each latent variable into three subgroups with the factor loading of each item. We aimed to balance the factor loading of each item parcel by pairing higher factor loaded items with lower factor loaded items (Seo, 2010). The goodness of fit of the proposed model was assessed using the Root Mean Squared Error Approximation (RMSEA) index, Comparative Fit Index (CFI), and Tucker-Lewis Index (TLI). Bootstrapping was used to examine the total and indirect effects (Shrout and Bolger, 2002). The parameters reported in the path analyses are obtained using maximum likelihood estimation method. Bootstrapping was used only to confirm the indirect and overall effects of the models tested.

## Results

Descriptive statistics and correlations between actual/ideal self-discrepancy, actual/ought self-discrepancy, shame, guilt, depression, and anxiety are shown in Table 1. All variables exhibited significant positive correlations, except for guilt and depression. Given that all variables were constructed with a single-factor structure, three observation variables were generated as sub-factors for each variable using the item parceling approach in preparation for testing the proposed model (Russell et al., 1998). To ensure the fulfillment of normality assumptions, skewness and kurtosis were assessed, and all variables met the relevant criteria (skewness ≤± 2, kurtosis ≤± 7; West et al., 1995).

**Table 1:** Descriptive statistics and scale intercorrelations.

	*M*	*SD*	1	2	3	4	5	6
1. Actual/ideal self-discrepancy	1.95	1.00	-					
2. Actual/ought self-discrepancy	1.81	0.93	0.75^***^	-				
3. Shame	2.89	0.69	0.37^***^	0.33^***^	-			
4. Guilt	3.78	0.52	0.32^***^	0.25^***^	0.32^***^	-		
5. Depression	1.08	0.58	0.39^***^	0.41^***^	0.48^***^	0.08	-	
6. Anxiety	2.39	0.62	0.38^***^	0.41^***^	0.47^***^	0.11^*^	0.77^***^	-

^*^p < 0.05, ^***^p < 0.001. *N* = 403.

Next, confirmatory factor analysis of the measurement models and an assessment of the proposed model’s fit were performed using the observed variables created through item parceling, following the two-step approach (Anderson and Gerbing, 1988). The results indicated a good model fit (CFI = 0.956, TLI = 0.944, RMSEA = 0.065, 90% CI: 0.057–0.074; Hu and Bentler, 1999). The factor loadings (β) of the observed variables ranged from 0.75 to 0.90, indicating a good factor loading state of over 0.50 (Hair et al., 2010). Having confirmed the fit of the measurement model in the initial stage, the theoretical relationship structure was further tested.

For the purpose of model comparison, we proposed two theoretical models: a partial mediation model, postulating direct effects of each actual/ideal self-discrepancy and actual/ought self-discrepancy on depression and anxiety, and a full mediation model, which does not assume any direct relationship between the two self-discrepancies and depression and anxiety. To determine which model better described the data, we assessed the fit indices of both proposed models. In addition, an AIC test (Akaike, 1973) was conducted along with a chi-squared difference test to compare the two nested models.

**Figure 1:**
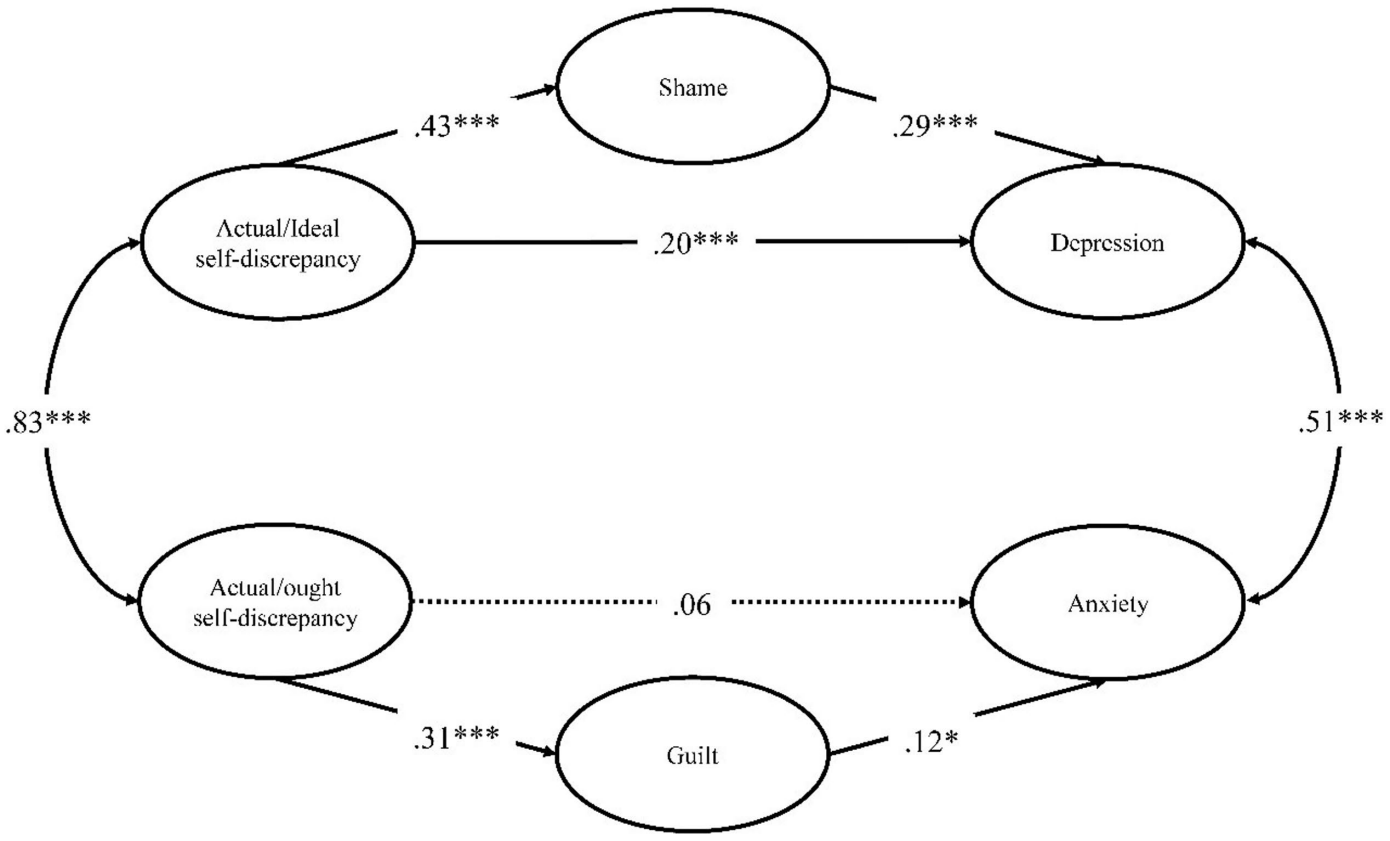
Standardized path coefficients of the final model (partial mediation model). ^*^p < 0.05, ^***^p < 0.001. Only the pathways between the latent and non-observed variables are presented. Statistically significant pathways are marked by solid lines and an insignificant pathway from actual/ought self-discrepancy to anxiety is marked by a dotted line.

The findings showed that both the partial and full mediation models showed a good fit (TLI > 0.90, CFI > 0.90, RMSEA < 0.08). The respective overall chi-squared values for the partial mediation model and the full mediation model were 401.843 and 414.008. When comparing the model fit between the nested models, a chi-square difference test revealed a significant difference between the two models. Consequently, the partial mediation model with additional direct pathways from the two self-discrepancies to depression and anxiety was selected as the final model ( $\Delta \chi^{2}{}_{partialmediationmodel-fullmediationmodel}\left(2\right)=12.165,p<0.01$ ). The path coefficients of the final model are presented in Table 2, and the standardized path coefficients and their significance are shown in Figure 1. Additionally, the AIC values for the partial and full mediation models were 525.840 and 534.008, respectively, indicating a better model fit for the partial mediation model.

**Table 2:** Regression coefficients of final model (partial mediation model).

Predictors			*B*	SE	*β*	*t*	$R^{2}$
Actual/ideal self-discrepancy	→	Shame	0.27	0.04	0.43	7.60^***^	0.184
Actual/ought self-discrepancy	→	Guilt	0.17	0.03	0.31	5.40^***^	0.096
Actual/ideal self-discrepancy	→	Depression	0.09	0.03	0.20	3.50^***^	0.175
Shame	→		0.21	0.04	0.29	5.18^***^	
Actual/ought self-discrepancy	→	Anxiety	0.03	0.02	0.06	1.11	0.023
Guilt	→		0.10	0.05	0.12	2.12^*^	
Actual/ideal self-discrepancy	↔	Actual/ought self-discrepancy	0.69	0.06	0.83	11.38^***^	-
Depression	↔	Anxiety	0.08	0.01	0.51	7.71^***^	

^*^p < 0.05, ^***^p < 0.001.

Regarding the mediating variables, shame was significantly positively associated with actual/ideal self-discrepancy ( β=0.43,t=7.60,p<0.001 ) and guilt was significantly positively associated with actual/ought self-discrepancy ( β=0.31,t=5.40,p<0.001 ). In relation to the criterion variables, depression was significantly positively associated with both actual/ideal self-discrepancy ( β=0.20,t=3.50,p<0.001 ) and shame ( β=0.29,t=5.18,p<0.001 ). However, anxiety was only significantly positively associated with guilt ( β=0.12,t=2.12,p<0.05 ) and not to actual/ought self-discrepancy. Next, residuals of actual/ideal- and actual/ought self-discrepancy ( β=0.83,t=11.38,p<0.001 ), and residuals of depression and anxiety ( β=0.51,t=7.71,p<0.001 ) were significantly positively correlated.

Finally, for the estimation of indirect effects and the calculation of 95% confidence intervals (CIs) for each effect, a bootstrapping procedure with 5,000 samples was conducted (Shrout and Bolger, 2002). This nonparametric resampling technique was utilized for the mediation analysis to determine indirect effects (Preacher and Hayes, 2008). The results of the bootstrapped mediation analyses are presented in Table 3. Notably, the indirect effect of actual/ideal self-discrepancy on depression through shame was statistically significant (B = 0.058, 95% Bias-corrected CI = 0.036~0.086), as was the indirect effect of actual/ought self-discrepancy on anxiety through guilt (B = 0.016, 95% Bias-corrected CI = 0.001~0.033). Therefore, the mediating effects of shame and guilt were unequivocally confirmed.

**Table 3:** Summary of results of bootstrapped mediation analyses.

Effect	Estimate	*SE*	95% confidence intervals (bias-corrected bootstrap)	
			Lower CI	Upper CI
Actual/ideal self-discrepancy → shame → depression	0.058	0.013	0.036	0.086
Actual/ought self-discrepancy → guilt → anxiety	0.016	0.008	0.001	0.033

Estimates are unstandardized. Size of bootstrap sample is 5,000.

## Discussion

We proposed two hypotheses grounded in Higgins (1987) self-discrepancy theory and Lewis (1987) findings regarding the distinctive functions of shame and guilt: shame mediates the relationship between actual/ideal self-discrepancy and depression (Hypothesis 1), while guilt mediates the relationship between actual/ought self-discrepancy and anxiety (Hypothesis 2). These hypotheses were supported empirically and theoretically. Despite the suggestion that two types of self-discrepancies could potentially predict depression and anxiety (Strauman and Higgins, 1988), existing research on self-discrepancy has not thoroughly illuminated the distinct mechanisms through which each type relates to specific psychological problems. We believe that much can be understood by fully examining self-discrepancy and psychological problems at the conceptual and theoretical levels. Accordingly, within an integrated single model, we empirically tested Higgins (1987) theoretical framework of self-discrepancy, identifying actual/ideal self-discrepancy and actual/ought self-discrepancy as discrete predictors of psychological vulnerabilities: actual/ideal self-discrepancy and actual/ought self-discrepancy being associated with depression and anxiety, respectively.

Furthermore, the mediating roles of shame and guilt in the relationship between the two self-discrepancies and psychological distress were substantiated. While previous studies have employed the self-discrepancy theory to explicate the distinct impacts of varying self-discrepancies on psychological maladjustment, some findings have exhibited variability (Tangney et al., 1998; Bruch et al., 2000). Boldero et al. (2005) suggested that an exploration of mediating or moderating variables in the self-discrepancy and psychological maladjustment relationship might be crucial. In line with this, the present study posited two distinct pathways through which actual/ideal self-discrepancy predicts depression, with shame as a mediator. Moreover, actual/ought self-discrepancy predicts anxiety with guilt as a mediator between the two variables.

### Limitations and directions for future research

The current study has several limitations. First, the sample included only college students in South Korea, so our findings may not be generalizable to different cultural regions, ages, or occupations. Therefore, future studies should collect data from different populations.

Second, we used self-report measures to assess self-discrepancy and subsequent emotions. A self-reported questionnaire can potentially provide socially desirable responses, resulting in bias. Future studies should implement multiple measurement methods to avoid possible biases in the results.

Third, the direct pathway between actual and ought self-discrepancy and anxiety was not statistically significant after guilt was included as a mediator. This suggests that potential moderator(s) affect the relationship between actual/ought self-discrepancy and anxiety. Indeed, Higgins (1999) argues that possible moderators are important when considering how different self-discrepancies relate to emotional vulnerabilities. For example, in one study, both emotion regulation and resilience moderated the relationship between ought self-discrepancy and anxiety (Gürcan-Yıldırım and Gençöz, 2020). In another study, self-acceptance was found to moderate the relationship between actual/ought self-discrepancy and interpersonal anxiety, suggesting that self-acceptance may play a protective role in shielding oneself from the negative ramifications of one’s perceived discrepancy regarding anxiety (Park and Chang, 2017). Future studies should focus on the proposed relationship between ought self-discrepancy and anxiety to further identify contributing factors.

Lastly, as our mediation models were tested using cross-sectional data, we could not determine causal relationships between the predictive variables and psychological outcomes. Longitudinal and experimental studies are needed to advance our understanding of self-discrepancy in relation to depression and anxiety.

## Data availability statement

The raw data supporting the conclusions of this article will be made available by the authors, without undue reservation.

## Author contributions

The research reported in the paper is derived from the first author’s master thesis submitted to Yonsei University under the supervision of the corresponding author. HO and D-gL contributed to the conception and design of the study. D-gL supervised and reviewed the manuscript. HC organized the database, performed the statistical analysis, and wrote the first draft of the manuscript. All authors contributed to the article and approved the submitted version.
