# Supplementary material for: The differential roles of shame and guilt in the relationship between self-discrepancy and psychological maladjustment
Source: Front Psychol. 2023 Sep 28;14:1215177. doi: 10.3389/fpsyg.2023.1215177 (PMC10573311; doi:10.3389/fpsyg.2023.1215177)
Supplement: Supplementary file 1 [file Data_Sheet_1.PDF]

Supplementary Material

1 Supplementary Data

We have further tested alternative models where shame and guilt have effects on both depression and anxiety. Specifically, a chi-squared difference test was conducted to compare the model fit indices. When comparing the original research model with the alternative model 1 where actual/ideal self-discrepancy is linked to depression and anxiety through shame, the chi-squared difference was insignificant to reject the alternative model. When comparing the original model with the alternative model 2 where actual/ought self-discrepancy is linked to anxiety and depression through guilt, the difference in chi-square values was significant (see table below). However, the mediating effect of guilt in the relationships between actual/ought self-discrepancy and each anxiety-depression was somewhat misleading, as the standardized path coefficient from guilt to anxiety was statistically insignificant and guilt was negatively associated with depression (see figure below). These findings are at odds with the theoretical explanation and previous literature we originally proposed (Higgins, 1987; Lewis, 1971). In line with suggestions from prior research (Boldero et al., 2005; Bruch et al., 2000), exploring a possible third variable to explain how guilt functions in predicting psychological distress from actual/ought self-discrepancy may be an important avenue for future research. In addition, these findings may be due to unique characteristics of the sample recruited in this study (e.g., emerging adulthood).

2 Supplementary Figures and Tables

2.1 Supplementary Figure

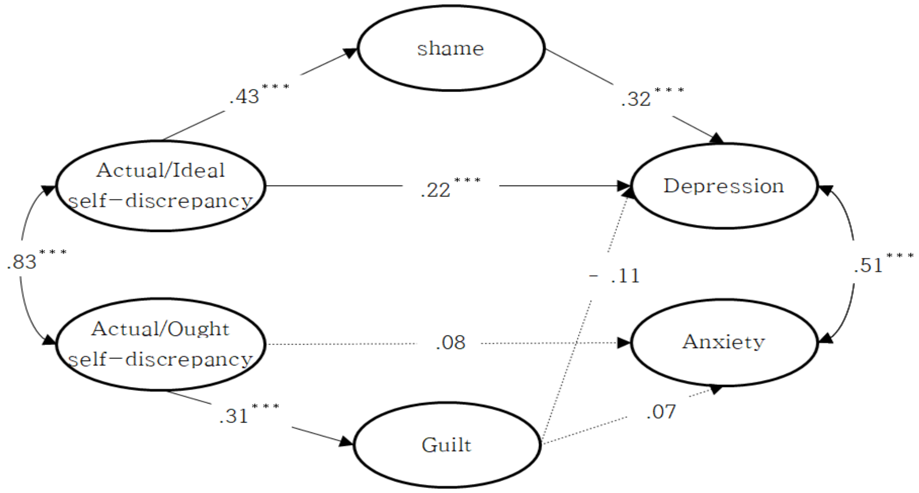

Supplementary Figure 1.

Standardized path coefficients of the alternative model 2.

2.2 Supplementary Table.

Supplementary Table 1.

Model fit indices and chi-squared difference test results

	χ^2	*df*	CFI	TLI	RMSEA (90% CI)
The Original Model	401.843	127	.941	.929	.073 (.065 - .082)
Alternative Model 2	398.230	126	.940	.930	.073 (.065 - .081)
Chi-square Difference Test	$\Delta\chi^2(1) = 3.613, p > .05$				

CI = Confidence Interval.
